# Evaluation of ABCA1 and FNDC3B Gene Polymorphisms Associated With Pseudoexfoliation Glaucoma and Primary Angle-Closure Glaucoma in a Saudi Cohort

**DOI:** 10.3389/fgene.2022.877174

**Published:** 2022-06-01

**Authors:** Altaf A. Kondkar, Tahira Sultan, Taif A. Azad, Essam A. Osman, Faisal A. Almobarak, Glenn P. Lobo, Saleh A. Al-Obeidan

**Affiliations:** ^1^ Department of Ophthalmology, College of Medicine, King Saud University, Riyadh, Saudi Arabia; ^2^ Glaucoma Research Chair in Ophthalmology, College of Medicine, King Saud University, Riyadh, Saudi Arabia; ^3^ King Saud University Medical City, King Saud University, Riyadh, Saudi Arabia; ^4^ Department of Ophthalmology and Visual Neurosciences, University of Minnesota, Minneapolis, MN, United States

**Keywords:** genetics, genotyping, glaucoma, intraocular pressure, ophthalmology, polymorphism, rs2472493, rs7636836

## Abstract

**Objective:** It is plausible that common disease mechanisms exist in glaucoma pathophysiology. Accordingly, we investigated the genetic association of two previously reported primary open-angle glaucoma (POAG)-related gene polymorphisms, rs2472493 (A > G) in *ABCA1* and rs7636836 (C > T) in FNDC3B, in primary angle-closure glaucoma (PACG) and pseudoexfoliation glaucoma (PXG).

**Methods:** TaqMan genotyping was performed in a total of 442 subjects consisting of 246 healthy controls, 102 PACG patients, and 94 PXG patients. Statistical evaluations were performed to detect allelic and genotype association of the variants with the disease and clinical variables such as intraocular pressure (IOP) and cup/disc ratio.

**Results:** Overall, there was no allelic or genotype association of these variants in PACG and PXG. However, rs7636836[T] allele significantly increased the risk of PXG among men (*p* = 0.029, odds ratio [OR] = 2.69, 95% confidence interval = 1.11–6.51). Similarly, rs2472493 and rs7636836 genotypes also showed significant association with PXG among men in over-dominant model (*p* = 0.031, OR = 1.98, 95% CI = 1.06–3.71) and co-dominant model (*p* = 0.029, OR = 2.69, 95% CI = 1.11–6.51), respectively. However, none survived Bonferroni’s correction. Besides, the synergic presence of rs2472493[G] and rs7636836[T] alleles (G-T) was found to significantly increase the risk of PACG (*p* = 0.026, OR = 2.85, 95% CI = 1.09–7.46). No significant genotype influence was observed on IOP and cup/disc ratio.

**Conclusion**: Our results suggest that the polymorphisms rs2472493 in ABCA1 and rs7636836 in FNDC3B genes may be associated with PXG among men, and a G-T allelic combination may confer an increased risk of PACG in the middle-eastern Saudi cohort. Further research in a larger population-based sample is needed to validate these findings.

## Introduction

Glaucoma is a complex multifactorial disease with high heritability and ethnic-specific predisposition, suggesting the involvement of genetic components in its pathogenesis (Chan et al., 2016). Among the various forms of glaucoma, primary angle-closure glaucoma (PACG) is a far more common type than primary open-angle glaucoma (POAG) in Asian populations, including Saudi Arabia (Al Obeidan et al., 2011; Chan et al., 2016) and involves anatomical blockage of the aqueous flow pathway (Weinreb et al., 2014). Likewise, pseudoexfoliation glaucoma (PXG) is the most severe type of open-angle glaucoma highly prevalent among the elderly, characterized by the abnormal deposition of pseudoexfoliative material (fibrillar extracellular matrix) in the anterior segment of the eye causing the aqueous blockage and associated with worse prognosis (Vazquez and Lee, 2014). Several disease-causing mutations and multiple susceptibility loci have been associated with different types of glaucoma in various ethnicities (Zukerman et al., 2021). However, the precise role of these genetic components and their underlying molecular mechanisms in glaucoma pathogenesis remains unclear.

Overlapping clinical manifestations among glaucoma types, such as high intraocular pressure (IOP), optic nerve damage, and retinal ganglion cell (RGC) apoptosis, suggest the plausibility of common disease pathways and genetic mechanisms in glaucoma pathogenesis. The previous genetic association studies of *MYOC* (Dai et al., 2008), *CYP1B1* (Chakrabarti et al., 2007), *LOXL1* (Shiga et al., 2018; Eliseeva et al., 2021), *ARHGEF12* (Aung et al., 2018), and *ACVR1* (Kondkar et al., 2020) that have been common to different types of glaucoma and therefore lend further support to this hypothesis. Besides, most of the studies investigating glaucoma genetics were conducted in Asian or Caucasian populations. Depending on the population being assessed, the same variants may or may not show an association. Therefore, replication studies must be performed to confirm the effects of these single nucleotide variants in other regions or with other ethnicities. The genetic basis of PACG and PXG in middle-eastern patients of Saudi origin is still largely unknown (Abu-Amero et al., 2010; Abu-Amero et al., 2013, 2015; Kondkar et al., 2021) and therefore warrants further genetic investigation. Accordingly, we hypothesized that genetic polymorphisms rs2472493 located upstream of the *ABCA1* gene and rs7636836 in *FNDC3B* were previously reported to be associated with POAG and elevated IOP (Hysi et al., 2014; Shiga et al., 2018), may also be associated with PACG and PXG in Saudi patients.

ABCA1 functions as a cholesterol efflux pump, and recent findings suggest its role in neuroinflammation, neurodegeneration, and RGC apoptosis (Howell et al., 2011; Awadalla et al., 2013). Whereas *FNDC3B* codes for an extracellular matrix protein involved in several signaling pathways including, transforming growth factor-beta (TGFβ) signaling (Prendes et al., 2013). Both genes are highly expressed in the human retina, optic nerve, trabecular meshwork, and RGC supporting its alleged role in glaucoma development and/or progression (Shiga et al., 2018). The genetic contribution of the variants at the *ABCA1* and *FNDC3B* locus among Saudi PACG and PXG patients is unknown. To date, there has been no study to address the relationship between the polymorphisms in *ABCA1* and *FNDC3B* in PACG and PXG in Saudi Arabs. Thus, in this exploratory study, we investigated whether single-nucleotide polymorphisms (SNPs) rs2472493 and rs7636836 upstream of *ABCA1* and in *FNDC3B* genes, respectively, were associated with PACG and PXG in a Saudi cohort.

## Materials and Methods

### Study Design and Population

The retrospective case-control study was approved by the Institutional Review Board Ethics Committee at the College of Medicine of King Saud University (IRB protocol approval number # 08–657) and adhered to the Declaration of Helsinki principles with all the participants providing written informed consent. Patients of Saudi origin with a clinical diagnosis of PACG, PXG, and non-glaucoma controls were recruited at King Abdulaziz University Hospital, Riyadh, Saudi Arabia from April 2017 through December 2019.

PACG patients (n = 102) were diagnosed based on clinical evidence of anatomically closed-angle showing the occurrence of appositional or synechial closure of the anterior chamber angle (at least 270° of the angle is occluded); raised IOP (≥21 mmHg); the presence of optic disk damage with cup/disc ratio of at least 0.7 (at least in one eye); and loss of peripheral or advanced visual field (Abu-Amero et al., 2013). PXG patients (n = 94) showed the presence of flaky exfoliation material along the pupil edges or anterior lens capsule, glaucomatous optic neuropathy and associated visual field loss, and high IOP in either or both the eyes as described previously (Kondkar et al., 2018). Patients harboring secondary forms of glaucoma, history of optic neuropathies or visual impairment unrelated to glaucoma, steroid usage, ocular trauma, absence of sufficient fundus visualization for disk assessment, or refusal to enroll were excluded. A group of healthy Saudi Arab participants (n = 246) recruited from our ophthalmology screening clinics served as controls. These participants were: >40 years of age, with normal IOP (<21 mmHg), open angles on gonioscopy, healthy optic disc (cup/disc ratio <0.5), free from any form of glaucoma on examination, and no family history of glaucoma. Subjects refusing to participate in the study were excluded.

### Genotyping of rs2472493 and rs7636836

Commercially available pre-designed TaqMan® assays, C__16235609_10 and C_189412462_10 purchased from Applied Biosystems (Catalog number: 4351379; Applied Biosystems Inc., Foster City, CA, United States) were used to genotype rs2472493 (A > G) and rs7636836 (C > T), respectively on ABI 7500 real-time PCR System (Applied Biosystems) according to the manufacturer instructions under recommended amplification conditions (Abu-Amero et al., 2013). Briefly, each assay utilizes two unlabeled PCR primers and two allele-specific probes. Each probe is labeled with a different color reporter dye (VIC® for allele 1 and 6-carboxy-fluorescein (FAM) for allele 2) at the 5′ end. The allele-specific fluorogenic probes when hybridized to the DNA template are cleaved by the 5′ nuclease activity of the Taq polymerase resulting in fluorescence emission from the reporter dye. Each PCR reaction was performed as recommended by the supplier in a total volume of 25 μl and consisted of 1X TaqMan® Genotyping Master Mix (Applied Biosystems), 1X SNP Genotyping Assay Mix and 20 ng DNA or molecular grade water in no template control well. The amplification conditions consisted of incubation at 95°C for 10 min, followed by 40 cycles, denaturation at 92°C for 15 s and annealing/ extension at 60°C for 1 min. The VIC® and FAM fluorescence levels of the PCR products were measured at 60°C for 1 min. Analysis of fluorescence using the automated 2-color allele discrimination software on ABI 7500 showed clear discrimination of all genotypes on a two-dimensional graph.

### Statistical Analysis

Hardy-Weinberg Equilibrium (HWE), gender distribution, allele and genotype associations were tested using Pearson’s Chi-square analysis and Fisher’s test where applicable. Normality testing of continuous variables was done using the Kolmogorov–Smirnov test. Accordingly, age differences and genotypes effects on glaucoma indices such as IOP, cup/disc ratio, and number of antiglaucoma medications were estimated using Mann-Whitney U test (2-groups comparison) and Kruskal–Wallis test (3-groups comparison). Binary logistic regression analysis was performed to test the effects of multiple factors (age, sex, genotypes) on glaucoma outcome. The analyses were performed using SPSS version 22 (IBM Inc., Chicago, Illinois, United States), Stat View software version 5.0 (SAS Institute, Cary, NC, United States), and SNPStats online software (https://www.snpstats.net/start.htm). The combined allelic (haplotype) effect was estimated using SHEsis (http://analysis.bio-x.cn/myAnalysis.php). Power analysis was done using Power Genetic Association (PGA) software (https://dceg.cancer.gov/tools/design/pga). A *p* < 0.05 (2-tailed) was considered significant. Bonferroni’s correction *p*-value for multiple testing was considered where applicable.

## Results

### Demographic Data Distribution

The demographic data of subjects included in the study is shown in Supplementary Figure S1. In comparison to the mean age of controls (59.5 years, ± 7.2), the mean age in PACG (60.6 years, ± 8.5) was not significantly different (*p* = 0.225), but the PXG cases (66.4 years, ± 9.7) were significantly older (*p* < 0.001). Besides, the frequency of gender distribution did not differ significantly in the PACG (*p* = 0.105) and PXG (*p* = 0.078) groups than in the controls.

### Allele Frequency of rs2472493 in *ABCA1* and rs7636836 in *FNDC3B* Genes

Of the total number of 442 subjects genotyped for rs2472493 and rs7636836 polymorphisms in this study, there were seven samples with missing genotypes for rs2472493 that failed to amplify and none for rs7636836, giving an estimated call rate of 98% and 100%, respectively. The samples with missing genotypes were excluded from rs2472493 and combined genotype analysis. Table 1 shows the minor allele frequency (MAF) distribution of rs2472493 and rs7636836 according to glaucoma types and gender in cases and controls. No significant deviation from HWE was observed (*p* > 0.05). Overall, the MAFs of both the polymorphisms showed no significant association with PACG and PXG. However, rs7636836 [T] variant in *FNDC3B* was significantly associated with increased risk of PXG among men (OR = 2.69, 95% CI = 1.11–6.51, *p* = 0.029). No such gender-specific association was observed for rs2472493 in *ABCA1*.

**TABLE 1:** Minor allele frequency of rs2472493 in *ABCA1* and rs7636836 in *FNDC3B* genes according to glaucoma types and gender.

Type variant	Cases MAF	Controls MAF	Odds ratio (95%confidence interval)	*p*-value
PACG				
rs2472493[G]				
Total	0.44	0.39	1.17 (0.86–1.61)	0.320
Men	0.42	0.37	1.21 (0.77–1.90)	0.420
Women	0.45	0.42	1.11 (0.71–1.73)	0.660
rs7636836[T]				
Total	0.05	0.05	1.10 (0.54–2.24)	0.790
Men	0.03	0.04	0.79 (0.21–2.95)	0.720
Women	0.07	0.06	1.21 (0.52–2.85)	0.660
PXG				
rs2472493[G]				
Total	0.37	0.39	0.89 (0.63–1.28)	0.554
Men	0.38	0.37	1.05 (0.67–1.65)	0.823
Women	0.34	0.42	0.72 (0.40–1.29)	0.269
rs7636836[T]				
Total	0.07	0.05	1.42 (0.72–2.80)	0.320
Men	0.10	0.04	**2.69 (1.11–6.51)**	**0.029**
Women	0.02	0.06	0.31 (0.04–2.21)	0.150

abbreviations: MAF, minor allele frequency; PACG, primary angle-closure glaucoma; PXG, pseudoexfoliation glaucoma.

Significant odds ratio and*p*-value in bold.

### Genotype Analysis of rs2472493 (*ABCA1*) and rs7636836 (*FNDC3B*) in PACG

Genotype association analysis was performed in co-dominant, dominant, recessive, over-dominant, and log-additive genetic models using SNPStats software. Both rs2472493 and rs7636836 genotypes did not show any significant association with PACG (Table 2). Furthermore, genotype analysis of both the variants in PACG did not reveal any gender-specific association in any of the tested genetic models (Supplementary Tables S1, S2).

**TABLE 2:** Association of rs2472493 (*ABCA1*) and rs7636836 (*FNDC3B*) polymorphisms with the risk of primary angle-closure glaucoma compared to control under different genetic models.

SNP number	Model	Genotype	Control n (%)	PACG n (%)	Odds ratio (95% confidence interval)	*p*-value	AIC	BIC	*p*-value^*^
rs2472493	Co-dominant	A/A	97 (39.6)	35 (35.0)	1.00	0.610	420.4	431.9	0.720
		G/A	104 (42.5)	43 (43.0)	1.15 (0.68–1.94)				
		G/G	44 (18.0)	22 (22.0)	1.39 (0.73–2.63)				
	Dominant	A/A	97 (39.6)	35 (35.0)	1.00	0.420	418.8	426.4	0.590
		G/A-G/G	148 (60.4)	65 (65.0)	1.22 (0.75–1.98)				
	Recessive	A/A-G/A	201 (82.0)	78 (78.0)	1.00	0.390	418.7	426.3	0.450
		G/G	44 (18.0)	22 (22.0)	1.29 (0.73–2.29)				
	Over-dominant	A/A-G/G	141 (57.5)	57 (57.0)	1.00	0.930	419.4	427.1	0.930
		G/A	104 (42.5)	43 (43.0)	1.02 (0.64–1.64)				
	Log-additive	---	---	---	1.17 (0.86–1.61)	0.320	418.4	426.1	0.440
rs7636836	Co-dominant	C/C	224 (91.1)	91 (89.2)	1.00	0.370	425.1	436.6	0.370
		C/T	20 (8.1)	11 (10.8)	1.35 (0.62–2.94)				
		T/T	2 (0.8)	0 (0)	0.00 (0.00-NA)				
	Dominant	C/C	224 (91.1)	91 (89.2)	1.00	0.600	424.7	432.4	0.660
		C/T-T/T	22 (8.9)	11 (10.8)	1.23 (0.57–2.64)				
	Recessive	C/C-C/T	244 (99.2)	102 (100.0)	1.00	0.240	423.6	431.3	0.220
		T/T	2 (0.8)	0 (0)	0.00 (0.00-NA)				
	Over-dominant	C/C-T/T	226 (91.9)	91 (89.2)	1.00	0.440	424.4	432.1	0.480
		C/T	20 (8.1)	11 (10.8)	1.37 (0.63–2.96)				
	Log-additive	---	---	---	1.10 (0.54–2.24)	0.790	424.9	432	0.870

*Adjusted for age and sex.

AIC, Akaike’s information criterion; BIC, Bayesian information criterion; PACG, primary angle-closure glaucoma.

### Genotype Analysis of rs2472493 (*ABCA1*) and rs7636836 (*FNDC3B*) in PXG

Overall, the polymorphisms rs2472493 and rs7636836 showed no significant association with PXG. However, a further gender stratification showed a significant moderate association of these polymorphisms with PXG among men (Tables 3, 4). Rs2472493 in *ABCA1* showed a significantly increased risk of PXG in men in the over-dominant model (OR = 1.98, 95% CI = 1.06–3.71, *p* = 0.031) that did not remain significant after adjustment for age, sex, and Bonferroni correction (Table 3). Similarly, although no homozygous rs7636836 [T/T] genotypes were observed in the PXG group, the heterozygous rs7636836 [C/T] genotype in *FNDC3B* showed significant association with PXG in men (OR = 2.69, 95% CI = 1.11–6.51, *p* = 0.029) in the co-dominant model that was significant after adjustment for age and sex (*p* = 0.026) but did not survive Bonferroni correction (Table 4).

**TABLE 3:** Genotype association analysis of polymorphism rs2472493 in *ABCA1* in pseudoexfoliation glaucoma.

Group	Genetic model	Genotype	Control n (%)	PXG n (%)	Odds ratio (95% confidence interval)	*p*-value	AIC	BIC	*p*-value^a^
Overall	Co-dominant	A/A	97 (39.6)	34 (37.8)	1.00	0.200	392.7	404.1	0.400
		G/A	104 (42.5)	46 (51.1)	1.26 (0.75–2.13)				
		G/G	44 (18.0)	10 (11.1)	0.65 (0.29–1.43)				
	Dominant	A/A	97 (39.6)	34 (37.8)	1.00	0.760	393.8	401.4	0.980
		G/A-G/G	148 (60.4)	56 (62.2)	1.08 (0.66–1.77)				
	Recessive	A/A-G/A	201 (82.0)	80 (88.9)	1.00	0.120	391.5	399.1	0.200
		G/G	44 (18.0)	10 (11.1)	0.57 (0.27–1.19)				
	Over-dominant	A/A-G/G	141 (57.5)	44 (48.9)	1.00	0.160	391.9	399.5	0.350
		G/A	104 (42.5)	46 (51.1)	1.42 (0.87–2.30)				
	Log-additive	---	---	---	0.90 (0.64–1.27)	0.560	393.5	401.2	0.530
Men	Co-dominant	A/A	56 (42.4)	19 (32.8)	1.00	0.079	234.7	244.5	0.150
		G/A	55 (41.7)	34 (58.6)	1.82 (0.93–3.57)				
		G/G	21 (15.9)	5 (8.6)	0.70 (0.23–2.12)				
	Dominant	A/A	56 (42.4)	19 (32.8)	1.00	0.210	236.2	242.7	0.460
		G/A-G/G	76 (57.6)	39 (67.2)	1.51 (0.79–2.89)				
	Recessive	A/A-G/A	111 (84.1)	53 (91.4)	1.00	0.160	235.8	242.3	0.140
		G/G	21 (15.9)	5 (8.6)	0.50 (0.18–1.40)				
	Over-dominant	A/A-G/G	77 (58.3)	24 (41.4)	1.00	**0.031**	233.1	239.6	0.086
		G/A	55 (41.7)	34 (58.6)	**1.98 (1.06–3.71)**				
	Log-additive	---	---	---	1.05 (0.67–1.65)	0.820	237.7	244.2	0.830
Women	Co-dominant	A/A	41 (36.3)	15 (46.9)	1.00	0.550	157.9	166.8	0.740
		G/A	49 (43.4)	12 (37.5)	0.67 (0.28–1.59)				
		G/G	23 (20.4)	5 (15.6)	0.59 (0.19–1.85)				
	Dominant	A/A	41 (36.3)	15 (46.9)	1.00	0.280	155.9	161.8	0.450
		G/A-G/G	72 (63.7)	17 (53.1)	0.65 (0.29–1.43)				
	Recessive	A/A-G/A	90 (79.7)	27 (84.4)	1.00	0.540	156.7	162.6	0.900
		G/G	23 (20.4)	5 (15.6)	0.72 (0.25–2.09)				
	Over-dominant	A/A-G/G	64 (56.6)	20 (62.5)	1.00	0.550	156.7	162.7	0.510
		G/A	49 (43.4)	12 (37.5)	0.78 (0.35–1.76)				
	Log-additive	---	---	---	0.75 (0.43–1.30)	0.290	156.0	161.9	0.560

*p-value adjusted for age and sex in the overall group and by age in men and women groups.

**Best-fit model *p*-value.

abbreviations: AIC, Akaike’s information criterion; BIC, Bayesian information criterion; OR (95% CI), Odds ratio (95% confidence interval); PXG, pseudoexfoliation glaucoma.

Significanct odds ratio and *p*-value in bold.

Bonnferoni corrected *p*-value is 0.01.

**TABLE 4:** Genotype association analysis of rs7636836 variant in *FNDC3B* in pseudoexfoliation glaucoma.

Group	Genetic model	Genotype	Control n (%)	PXG n (%)	Odds ratio (95% confidence interval)	*p*-value	AIC	BIC	*p*-value^*^
Overall	Co-dominant	C/C	224 (91.1)	81 (86.2)	1.00	0.170	403.3	414.8	0.280
		C/T	20 (8.1)	13 (13.8)	1.80 (0.85–3.78)				
		T/T	2 (0.8)	0 (0)	0.00 (0.00-NA)				
	Dominant	C/C	224 (91.1)	81 (86.2)	1.00	0.200	403.3	410.9	0.190
		C/T-T/T	22 (8.9)	13 (13.8)	1.63 (0.79–3.40)				
	Recessive	C/C-C/T	244 (99.2)	94 (100.0)	1.00	0.250	403.6	411.3	0.460
		T/T	2 (0.8)	0 (0)	0.00 (0.00-NA)				
	Over-dominant	C/C-T/T	226 (91.9)	81 (86.2)	1.00	0.120	402.6	410.2	0.150
		C/T	20 (8.1)	13 (13.8)	1.81 (0.86–3.81)				
	Log-additive	---	---	---	1.42 (0.72–2.80)	0.320	403.9	411.6	0.270
Men	--	C/C	121 (91.7)	49 (80.3)	1.00	**0.029**	240.0	246.6	**0.026**
		C/T	11 (8.3)	12 (19.7)	**2.69 (1.11–6.51)**				
		T/T	0	0	-				
Women	Co-dominant	C/C	103 (90.3)	32 (97.0)	1.00	0.330	160.4	169.3	0.500
		C/T	9 (7.9)	1 (3.0)	0.36 (0.04–2.93)				
		T/T	2 (1.8)	0 (0)	0.00 (0.00-NA)				
	Dominant	C/C	103 (90.3)	32 (97.0)	1.00	0.180	158.7	164.7	0.270
		C/T-T/T	11 (9.7)	1 (3.0)	0.29 (0.04–2.35)				
	Recessive	C/C-C/T	112 (98.2)	33 (100.0)	1.00	0.310	159.5	165.5	0.490
		T/T	2 (1.8)	0 (0)	0.00 (0.00-NA)				
	Over-dominant	C/C-T/T	105 (92.1)	32 (97.0)	1.00	0.290	159.4	165.4	0.350
		C/T	9 (7.9)	1 (3.0)	0.36 (0.04–2.99)				
	Log-additive	---	---	---	0.31 (0.04–2.21)	0.150	158.5	164.5	0.250

*p-value adjusted for age and sex in the overall group and by age in men and women groups.

Note: Significant odds ratio and *p*-value in bold. Bonferroni corrected *p*-value is 0.01.

abbreviations: AIC, Akaike’s information criterion; BIC, Bayesian information criterion; PXG, pseudoexfoliation glaucoma.

### Combined Genotype and Allele Frequency Analysis in PACG and PXG

The combined genotype analysis of the *ABCA1* rs2472493 (A > G) and *FNDC3B* rs7636836 (C > T) polymorphisms did not show any significant effect on PACG susceptibility but did suggest that the presence of GG-CT genotype would increase the risk of PACG by 2.5-fold (OR = 2.55, 95% CI = 0.49–13.30), albeit non-significantly (*p* = 0.356) (Supplementary Table S3). In addition, the analysis of synergic effects of *ABCA1* rs2472493 and *FNDC3B* rs7636836 alleles showed that the synergic presence of rs2472493[G] and rs7636836[T] alleles (G-T) could lead to a significantly (*p* = 0.026) increased risk of PACG (OR = 2.85, 95% CI = 1.09–7.46) (Supplementary Table S4). The analysis, however, did not account for multiple testing corrections. In contrast, combined genotype and allele frequency analysis in PXG yielded no significant result (Supplementary Tables S5, S6).

### Regression Analysis and Effect of Genotypes on Clinical Indices of Glaucoma

A binary logistic regression analysis was performed to detect the effect of age, sex, and polymorphisms rs2472493 in *ABCA1* and rs7636836 in *FNDC3B* on disease outcomes. None of these factors were found to have any significant effect on PACG and PXG outcomes. However, age remained a significant predictor of PXG (Supplementary Table S7). Furthermore, the genotype effect of rs2472493 (*ABCA1*) and rs7636836 (*FNDC3B*) polymorphisms on IOP, cup/disc ratio, and the number of antiglaucoma medications in the PACG and PXG patient groups were also examined. These phenotypes are clinical indicators related to disease severity. The analysis revealed no significant genotype effect on any of these clinical markers in both the PACG and PXG patient groups (Supplementary Figure S2).

## Discussion

Glaucoma is a complex polygenic disorder affected by multiple genetic and environmental factors. Previously, several genome-wide association studies have identified numerous genetic polymorphisms and susceptibility loci in different populations worldwide, few of which have been unique to specific ethnic groups (Zukerman et al., 2021). However, the genetic etiology of glaucoma in middle-eastern population is still lacking and therefore warrants further studies. Given the overlapping clinical manifestations and common genetic variants reported among glaucoma types, we investigated the association of two POAG-related gene polymorphisms, rs2472493 in *ABCA1* gene and rs7636836 near *FNDC3B*, in the PACG and PXG patient cohort of Saudi origin.

In our study, the MAF of rs2472493 in *ABCA1* was 0.44 and 0.37 in PACG and PXG, respectively, and were comparable to those in controls (0.39) and thus non-significant. The allele frequencies were similar to those reported in the Han Chinese and Uygur Chinese population of PACG (0.45) and PXG (0.40) patients, respectively (Luo et al., 2015). Likewise, the MAFs were comparable to the Hispanics (0.37), non-Hispanics (0.42), and African American (0.35) POAG subjects (Choquet et al., 2018), but lower than the East-Asians (0.56) (Choquet et al., 2018) and the European (0.51) POAG patients (Gharahkhani et al., 2014) highlighting slight ethnic and glaucoma type variability.

Previously, SNP rs2472493 in *ABCA1* was associated with POAG in the genome-wide meta-analysis of 18 population cohorts from the International Glaucoma Genetics Consortium (Hysi et al., 2014). Similar genome-wide findings were reported by Gharahkhani et al., in the Australian cohort (Gharahkhani et al., 2014) and by Chen et al., in POAG patients from Southern China (Chen et al., 2014). Thus, multiple population studies indicated that the ABCA1 gene might play a crucial role in POAG pathogenesis. In contrast, negative *ABCA1* gene associations have been reported in the Han Chinese cohort of 1,311 healthy controls and 1122 PACG patients (Luo et al., 2015), and in the 52 Jordanian Arab glaucoma patients (POAG and congenital) and 96 healthy individuals investigated for the *ABCA1* rs2472493 variant by Alkhatib et al. (2019). Although our study did not replicate the findings in the PXG and PACG patient cohort of Saudi origin, we observed a significant association of *ABCA1* rs2472493 polymorphism among men in the PXG cohort. However, there are no published reports of this variant being examined in PXG.

ABCA1 belongs to a large superfamily of ABC transmembrane transporters and is best known for its role in cholesterol efflux to lipid-free apolipoprotein AI and apolipoprotein E (Wang and Smith, 2014). ABCA1 is expressed in all the major tissues of the eye, including the trabecular meshwork, Schlemm’s canal endothelial cells, RGCs, and optic nerve that are primarily involved in glaucoma (Chen et al., 2014). However, the exact mechanism(s) by which ABCA1 may be involved in glaucoma pathogenesis is still unclear. ABCA1 was demonstrated to inhibit ocular inflammation via activation of liver X-receptor in an experimental model of autoimmune uveitis (Yang et al., 2014). Using the glaucoma model, Li et al. demonstrated that ABCA1 was related to RGC death (Li et al., 2018). These studies suggest that ABCA1 may have a significant role in eye research. Likewise, using the Encyclopedia of DNA Elements (ENCODE) project data and Genevar database, Gharahkhani and colleagues, reported that the polymorphism rs2472493 located upstream of *ABCA1* is an expression quantitative trait locus (eQTL) in lymphoblastoid cell lines that might alter the motif sequences for proteins such as FOXJ2 and SIX5 (Gharahkhani et al., 2014). Also, rs2472493 was found to be in high linkage disequilibrium with polymorphism rs2472494 near *ABCA1* that alters the regulatory motif for binding of *PAX6*, a gene involved in eye development. The authors thereby predicted a possible regulatory role for this polymorphism in gene expression in a pathway similar to that of rs2472494 variant near *ABCA1* gene (Gharahkhani et al., 2014). However, the exact mechanism(s) by which rs2472493 polymorphism in *ABCA1* might increase PXG risk in men is unknown. The effect of gender, gene-gene, and gene-environment interactions on *ABCA1* gene polymorphisms in lipid and lipoprotein metabolism have been well documented (Junyent et al., 2010; Coban et al., 2014; Shi et al., 2021). The *ABCA1* polymorphism might plausibly modulate the risk of PXG among men in a similar manner.

*FNDC3B* (also known as *FAD104*) is a known regulator of adipogenesis (Tominaga et al., 2004). *FNDC3B* codes for an extracellular matrix protein that has a vital role in cell adhesion and growth signaling pathways, including TGFβ, and Wnt/β-catenin signaling (Nishizuka et al., 2009; Goto et al., 2017; Li et al., 2020) all of which have been strongly implicated in glaucoma pathogenesis (Prendes et al., 2013; Zhong et al., 2013; Webber et al., 2018). Furthermore, *FNDC3B* is expressed in all the primary eye tissues relevant to glaucoma (Li et al., 2015; Shiga et al., 2018).

The variant rs7636836 in *FNDC3B* was one of the novel loci associated with POAG in a two-stage genome-wide study consisting of 7,378 Japanese POAG cases and 36,385 controls conducted by Shiga et al. (2018). However, further validation in Singapore Chinese, European, and Africans did not show any significant association (Shiga et al., 2018). In our study cohort, the MAF of rs7636836 in *FNDC3B* was 0.05 and 0.07 in PACG and PXG, respectively. The frequency distribution was similar to European (0.06) but lower than the Japanese (0.40) and Singaporean Chinese (0.21) POAG subjects reported by Shiga et al. (2018). Our data showed no significant allelic and genotype association between rs7636836 in *FNDC3B* and PACG/PXG. However, in men, a modest allelic and genotype association of rs7636836 in *FNDC3B* was observed in PXG. The potential mechanism(s) by which rs7636836 in *FNDC3B* would modulate the risk of PXG in men is unclear but could possibly be hormonal (androgen) related via Wnt signaling which can be stimulated by *FNDC3B* (Li et al., 2020). There is evidence for crosstalk between androgen receptor and Wnt/ß-catenin signaling pathway (Mumford et al., 2018). The androgen receptors are expressed in ocular tissues (Tachibana et al., 2000) and Wnt signaling is strongly implicated in the maintenance of glaucomatous trabecular meshwork (Dhamodaran et al., 2020). To the best of our knowledge, there are no published reports of studies evaluating this polymorphism in PACG and PXG patients.

Gene-gene and gene-environmental interactions have been suggested to play an essential role in the pathogenesis of complex human diseases and highlight the possible contribution of genetic background and environmental triggers in disease development and progression (Marchini et al., 2005; Pan, 2008; Brossard et al., 2015). Glaucoma is also a complex polygenic disease with no clear inheritance pattern, and similar mechanisms may exist to influence the disease outcomes (Zakharov et al., 2013). Interestingly, a combined allelic and genotype analysis of rs2472493 in *ABCA1* and rs7636836 in *FNDC3B* showed that G-T allele haplotype of *ABCA1* and *FNDC3B* was associated with a significantly increased risk of PACG. Although it is difficult to ascertain whether the risk observed in our study is attributable to a real haplotype effect or probably reflects a linkage with other variant(s) not included in this study, the possible role of rs2472493 in *ABCA1* and rs7636836 in *FNDC3B* in PACG cannot be completely ruled out and would need further research in a larger cohort.

Polymorphisms in *ABCA1* and *FNDC3B* have also been shown to influence IOP (Hysi et al., 2014). A recent study showed that ABCA1 regulated IOP by modulating caveolin-1, nitric oxide/endothelial nitric oxide synthase signaling pathway (Hu et al., 2020). However, the analysis of polymorphisms effect on clinical endophenotypes of PACG and PXG, such as IOP and cup/disc ratio in our cohort, showed no significant association.

Thus, our results show that rs2472493 in *ABCA1* and rs7636836 in *FNDC3B* may not have a major direct role in PACG and PXG in this ethnic group. However, the association of rs7636836 in *FNDC3B* and rs2472493 in *ABCA1* observed among PXG men; and that of G-T haplotype in PACG suggests that they may have a significant indirect role (possibly via epistatic interaction(s)) in PXG and PACG. But since the study was performed in a relatively small sample size and does not provide any mechanistic evidence, the results would require a cautious interpretation. Based on the observed allele frequencies, the study had an estimated power of >0.9 per allele for the *ABCA1* variant but was underpowered (0.6 per allele) for the *FNDC3B* variant to detect a relative risk of 2.0 with an alpha type I error of 0.05. Thus, the possibility of chance association cannot be ruled out and further emphasizes the need for replication in a large sample cohort potentially with age and gender-matched controls to confirm these findings.

In conclusion, our results suggest that the polymorphisms rs2472493 in ABCA1 and rs7636836 in FNDC3B genes may be associated with PXG among men, and a G-T allelic combination may confer an increased risk of PACG in the middle-eastern Saudi cohort. However, further investigations in larger population-based samples and different ethnicity are needed to draw definite conclusions and validate these findings. Moreover, considering the genetic heterogeneity of glaucoma *per se*, the plausible involvement of gene-gene and/or gene–environmental interactions must be important considerations for future research.

## Data Availability

The original contributions presented in the study are included in the article/Supplementary Material, further inquiries can be directed to the corresponding author.
