# Supplementary material for: Evaluation of ABCA1 and FNDC3B Gene Polymorphisms Associated With Pseudoexfoliation Glaucoma and Primary Angle-Closure Glaucoma in a Saudi Cohort
Source: Front Genet. 2022 Jun 1;13:877174. doi: 10.3389/fgene.2022.877174 (PMC9198278; doi:10.3389/fgene.2022.877174)
Supplement: Supplementary file 1 [file DataSheet1.PDF]

Fig. S1. Demographic data of patients and controls

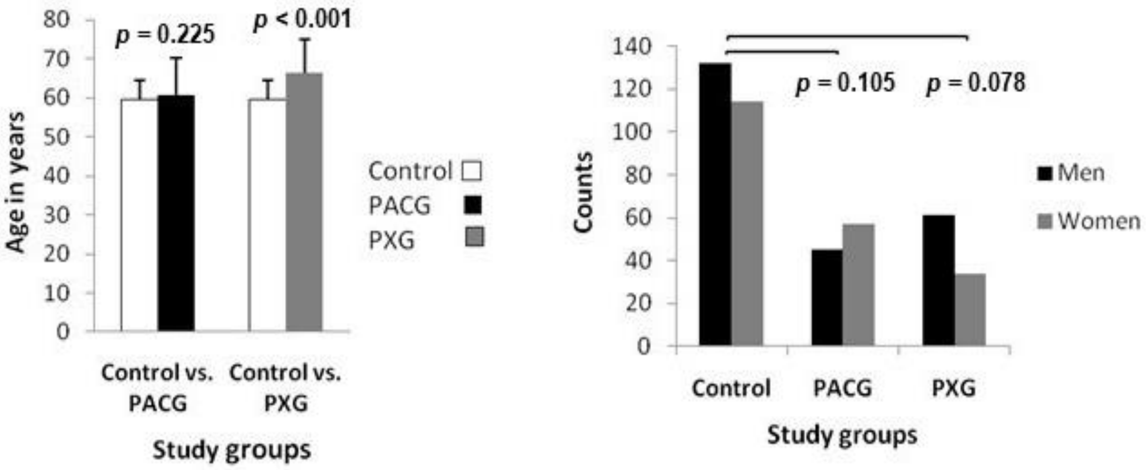

Abbreviation: PACG, primary angle-closure glaucoma; PXG – pseudoexfoliation glaucoma.

Fig. S2. Genotype effects of polymorphisms (A) rs2472493 and (B) rs7636836 variants on intraocular pressure, cup/disc ratio, and the number of antiglaucoma medication in primary angle-closure glaucoma (PACG) and pseudoexfoliation glaucoma (PXG) patients.

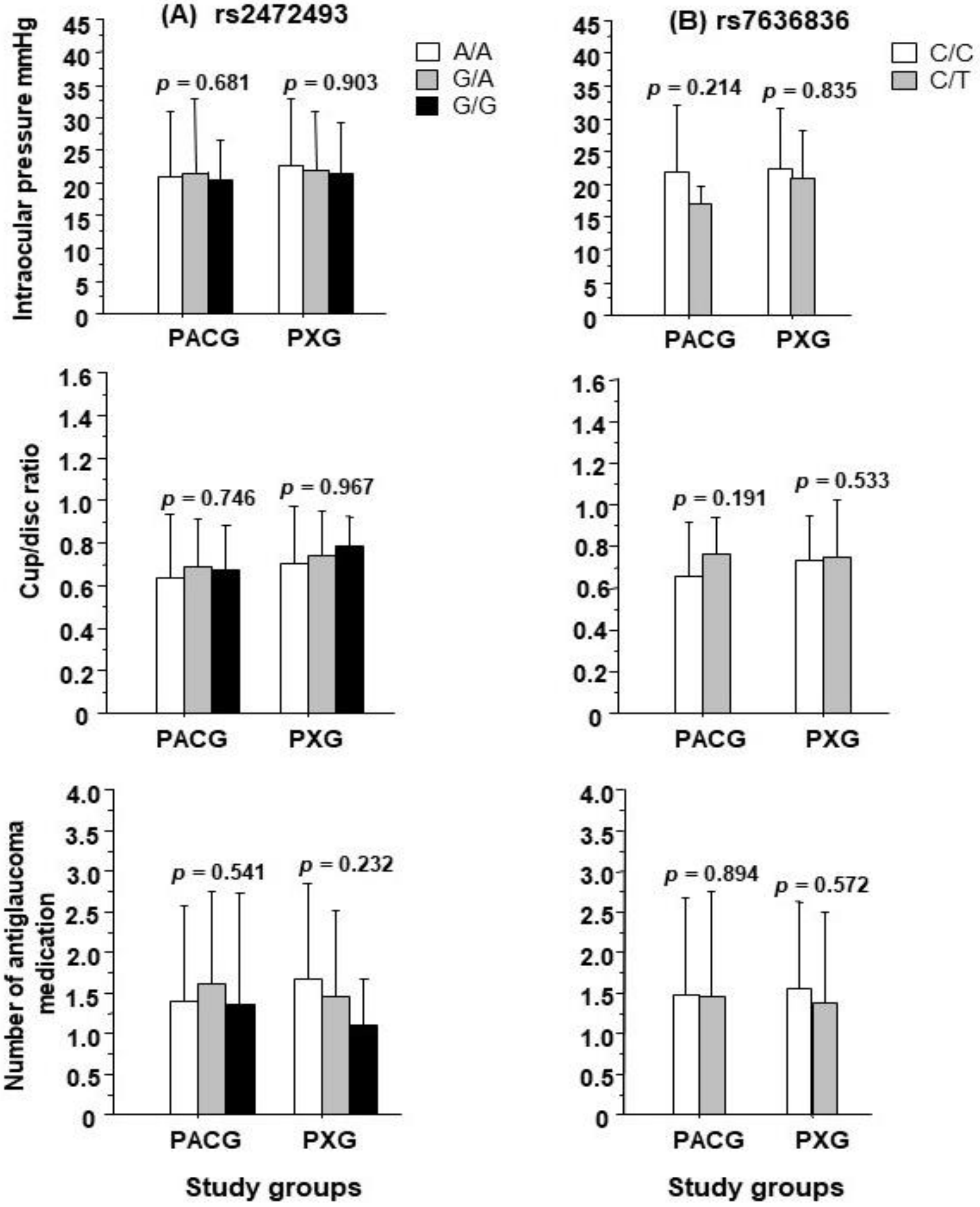

Note: No T/T homozygous were observed for rs7636836.

Table S1. Gender-stratified association analysis of rs2472493 polymorphism in *ABCA1* with primary angle-closure glaucoma

Group	Genetic Model	Genotype	Control n (%)	PACG n (%)	Odds ratio (95% confidence interval)	*p*-value	AIC	BIC	*p*-value§
Men	Co-dominant	A/A	56 (42.4)	19 (43.2)	1.00				
		G/A	55 (41.7)	13 (29.6)	0.70 (0.31-1.55)	0.180	200.5	210	0.190
		G/G	21 (15.9)	12 (27.3)	1.68 (0.70-4.06)				
	Dominant	A/A	56 (42.4)	19 (43.2)	1.00	0.930	201.9	208.3	0.820
		G/A-G/G	76 (57.6)	25 (56.8)	0.97 (0.49-1.93)				
	Recessive	A/A-G/A	111 (84.1)	32 (72.7)	1.00	0.100	199.3	205.7	0.120
		G/G	21 (15.9)	12 (27.3)	1.98 (0.88-4.46)				
	Over-dominant	A/A-G/G	77 (58.3)	31 (70.5)	1.00	0.150	199.8	206.2	0.130
		G/A	55 (41.7)	13 (29.6)	0.59 (0.28-1.22)				
	Log-additive	---	---	---	1.21 (0.77-1.90)	0.420	201.3	207.6	0.490
Women	Co-dominant	A/A	41 (36.3)	16 (28.6)	1.00				
		G/A	49 (43.4)	30 (53.6)	1.57 (0.75-3.27)	0.450	219.1	228.4	0.430
		G/G	23 (20.4)	10 (17.9)	1.11 (0.43-2.85)				
	Dominant	A/A	41 (36.3)	16 (28.6)	1.00	0.310	217.7	223.9	0.320
		G/A-G/G	72 (63.7)	40 (71.4)	1.42 (0.71-2.85)				
	Recessive	A/A-G/A	90 (79.7)	46 (82.1)	1.00	0.700	218.5	224.8	0.670
		G/G	23 (20.4)	10 (17.9)	0.85 (0.37-1.94)				
	Over-dominant	A/A-G/G	64 (56.6)	26 (46.4)	1.00	0.210	217.1	223.4	0.200
		G/A	49 (43.4)	30 (53.6)	1.51 (0.79-2.87)				
	Log-additive	---	---	---	1.11 (0.71-1.73)	0.660	218.5	224.7	0.670

§*p*-value adjusted for age and sex in overall group and by age in men and women groups

Abbreviations: AIC, Akaike's information criterion; BIC, Bayesian information criterion; PACG primary angle-closure glaucoma.

Table S2. Gender-stratified association analysis of rs7636836 polymorphism in *FNDC3B* with primary angle-closure glaucoma

Group	Genetic Model	Genotype	Control n (%)	PACG n (%)	OR (95% CI)	*p*-value	AIC	BIC	*p*-value§
Men	--	C/C	121 (91.7)	42 (93.3)	1.00	0.720	204.6	210.9	0.740
		C/T	11 (8.3)	3 (6.7)	0.79 (0.21-2.95)				
		T/T	0 (0)	0 (0)	-				
Women	Co-dominant	C/C	103 (90.3)	49 (86.0)	1.00	0.220	220.6	230.0	0.250
		C/T	9 (7.9)	8 (14.0)	1.87 (0.68-5.14)				
		T/T	2 (1.8)	0 (0)	0.00 (0.00-NA)				
	Dominant	C/C	103 (90.3)	49 (86.0)	1.00	0.400	221.0	227.3	0.430
		C/T-T/T	11 (9.7)	8 (14.0)	1.53 (0.58-4.04)				
	Recessive	C/C-C/T	112 (98.2)	57 (100.0)	1.00	0.200	220.1	226.3	0.220
		T/T	2 (1.8)	0 (0)	0.00 (0.00-NA)				
	Over-dominant	C/C-T/T	105 (92.1)	49 (86.0)	1.00	0.220	220.2	226.4	0.250
		C/T	9 (7.9)	8 (14.0)	1.90 (0.69-5.23)				
	Log-additive	---	---	---	1.21 (0.52-2.85)	0.660	221.5	227.8	0.670

§*p*-value adjusted for age and sex in overall group and by age in men and women groups

Abbreviations: AIC, Akaike's information criterion; BIC, Bayesian information criterion; PACG, primary angle-closure glaucoma.

Note: No homozygous rs7636836 T/T genotypes were observed among men.

Table S3. Combined genotype effects of *ABCA1* rs2472493 and *FNDC3B* rs7636836 polymorphisms in primary angle-closure glaucoma

Rs2472493	Rs7636836	Controls n (%)	PACG n (%)	Fisher's *p*	Odds ratio (95% Confidence interval)
AA	CC	87 (35.5)	34 (34.0)	-	Reference
AA	CT	10 (4.0)	1 (1.0)	0.286	-
AA	TT	0 (0)	0 (0)	-	-
AG	CC	95 (38.7)	36 (36.0)	0.920	0.97 (0.55-1.68)
AG	CT	7 (2.8)	7 (7.0)	0.123	2.55 (0.83-7.84)
AG	TT	2 (0.8)	0 (0)	1.000	-
GG	CC	41 (16.7)	19 (19.0)	0.728	1.18 (0.60-2.32)
GG	CT	3 (1.2)	3 (3.0)	0.356	2.55 (0.49-13.30)
GG	TT	0 (0)	0 (0)	-	-
AA	CC	87 (35.5)	34 (34.0)	-	Reference
Other genotypes		158 (64.4)	66 (66.0)	0.805	1.06 (0.65-1.74)

PACG, primary angle-closure glaucoma

Table S4. Combination effects of *ABCA1* rs2472493 and *FNDC3B* rs7636836 alleles in primary angle-closure glaucoma

Allele combination*	PACG Frequency	Controls Frequency	Fisher's $p^{\dagger}$	Odds ratio (95% Confidence interval)
A-C	0.556	0.576	0.628	0.921 (0.661~1.284)
A-T	0.009	0.033	0.083	0.285 (0.063~1.295)
G-C	0.389	0.375	0.729	1.061 (0.757~1.488)
G-T	0.046	0.016	**0.026**	2.851 (1.089~7.466)

*In the order of rs247243 and rs7636836.

†Uncorrected *p*-value.

Overall Chi^2 = 7.92, df = 3, Fisher's *p*-value = **0.047**. Significant *p*-value in bold.

PACG, primary angle-closure glaucoma

Table S5. Combined genotype effects of *ABCA1* rs247243 and *FNDC3B* rs7636836 polymorphisms on risk of pseudoexfoliation glaucoma

Rs2472493	Rs7636836	Controls n (%)	PXG n (%)	Fisher's *p*	Odds ratio (95% Confidence interval)
AA	CC	87 (35.5)	27 (30.3)	-	Reference
AA	CT	10 (4.0)	6 (6.7)	0.356	1.93 (0.64-5.81)
AA	TT	0 (0)	0 (0)	-	-
AG	CC	95 (38.7)	40 (44.9)	0.317	1.35 (0.76-2.39)
AG	CT	7 (2.8)	6 (6.7)	0.098	2.76 (0.85-8.92)
AG	TT	2 (0.8)	0 (0)	-	-
GG	CC	41 (16.7)	9 (10.1)	0.539	0.70 (0.30-1.64)
GG	CT	3 (1.2)	1 (1.1)	1.00	1.07 (0.10-10.75)
GG	TT	0 (0)	0 (0)	-	-
AA	CC	87 (35.5)	27 (30.3)	-	Reference
Other genotypes		158 (64.4)	62 (69.6)	0.434	1.20 (0.75-2.13)

PXG, pseudoexfoliation glaucoma

Table S6. Combination effects of *ABCA1* rs2472493 and *FNDC3B* rs7636836 alleles in pseudoexfoliation glaucoma

Allele combination*	PXG Frequency	Controls Frequency	Fisher's $p^{\dagger}$	Odds ratio (95% Confidence interval)
A-C	0.573	0.576	0.943	0.987 (0.698~1.397)
A-T	0.057	0.033	0.154	1.787 (0.796~4.008)
G-C	0.354	0.375	0.619	0.913 (0.639~1.306)
G-T	0.016	0.016	0.994	0.995 (0.258~3.841)

*In the order of rs2472493 and rs7636836.

†Uncorrected *p*-value.

Overall Chi^2 = 2.11, df = 3, Fisher's *p*-value = 0.549.

PXG, pseudoexfoliation glaucoma

Table S7. Binary logistic regression analysis to determine the effect age, sex and polymorphisms on glaucoma risk

Group Variables	B	S.E.	Wald	*p*-value	Odds ratio (95% confidence interval)
**PACG**					
Age	0.021	0.016	1.667	0.197	1.021 (0.9-1.05)
Sex	-0.420	0.242	3.016	0.082	0.657 (0.41-1.05)
Rs2472493			0.635	0.728	
G/A	0.091	0.272	0.112	0.738	1.095 (0.64-1.86)
G/G	0.263	0.331	0.634	0.426	1.301 (0.68-2.49)
Rs7636836			0.512	0.774	
C/T	0.286	0.400	0.512	0.474	1.331 (0.60-2.91)
T/T	--	--	--	--	--
Constant	-2.042	.974	4.397	0.036	0.130
**PXG**					
Age	0.101	0.017	36.649	**0.000**	1.107 (1.07-1.14)
Sex	0.313	0.275	1.301	0.254	1.368 (0.79-2.34)
Rs2472493			1.265	0.531	
G/A	0.138	0.289	0.227	0.634	1.148 (0.65-2.02)
G/G	-0.318	0.419	0.577	0.448	0.728 (0.32-1.65)
Rs7636836			2.319	0.314	
C/T	0.645	0.424	2.319	0.128	1.907 (0.83-4.37)
T/T	--	--	--	--	--
Constant	-7.619	1.100	48.011	0.000	0.000

Abbreviations: PACG primary angle-closure glaucoma, PXG pseudoexfoliation glaucoma
Note: Significant *p*-value in bold
